# Does MIS-TLIF or TLIF result in better pedicle screw placement accuracy and clinical outcomes with navigation guidance?

**DOI:** 10.1186/s12891-022-05106-1

**Published:** 2022-02-16

**Authors:** Jia Bin Liu, Jun Long Wu, Rui Zuo, Chang Qing Li, Chao Zhang, Yue Zhou

**Affiliations:** 1 Department of Orthopaedics, Xinqiao Hospital, Amy Medical University (Third Military Medical University), Chongqing, 400037 People’s Republic of China; 2 Department of Orthopaedics, The Hospital of People Liberation Army Hong Kong Garrison, Hong Kong, 999077 People’s Republic of China; 3 Department of Orthopaedics, The 941 Hospital of Chinese People Liberation Army, Xining, 810007 People’s Republic of China

**Keywords:** Computer-assisted navigation, Minimally invasive surgery, Pedicle screw implantation, Spine surgery

## Abstract

**Background:**

Although previous studies have suggested that navigation can improve the accuracy of pedicle screw placement, few studies have compared navigation-assisted transforaminal lumbar interbody fusion (TLIF) and navigation-assisted minimally invasive TLIF (MIS-TLIF). The entry point of pedicle screw insertion in navigation-assisted MIS-TLIF (NM-TLIF) may deviate from the planned entry point due to an uneven bone surface, which may result in misplacement. The purpose of this study was to explore the pedicle screw accuracy and clinical consequences of MIS-TLIF and TLIF, both under O-arm navigation, to determine which surgical method is better.

**Methods:**

A retrospective study of 54 patients who underwent single-segment NM-TLIF or navigation-assisted TLIF (N-TLIF) was conducted. In addition to the patients’ demographic characteristics, intraoperative indicators and complications, the Oswestry Disability Index (ODI) and visual analog scale (VAS) score were recorded and analyzed preoperatively and at the 1-, 6-, and 12-month and final postoperative follow-ups. The clinical qualitative accuracy and absolute quantitative accuracy of pedicle screw placement were assessed by postoperative CT. Multifidus muscle injury was evaluated by T2-weighted MRI.

**Results:**

Compared with N-TLIF, NM-TLIF was more advantageous in terms of the incision length, intraoperative blood loss, drainage volume, time to ambulation, length of hospital stay, blood transfusion rate and analgesia rate (*P* < 0.05). The ODI and VAS scores for low back pain were better than those of N-TLIF at 1 month and 6 months post-surgery (*P* < 0.05). There was no significant difference in the clinical qualitative screw placement accuracy (97.3% vs. 96.2%, *P* > 0.05). The absolute quantitative accuracy results showed that the axial translational error, sagittal translational error, and sagittal angle error in the NM-TLIF group were significantly greater than those in the N-TLIF group (*P* < 0.05). The mean T2-weighted signal intensity of the multifidus muscle in the NM-TLIF group was significantly lower than that in the N-TLIF group (*P* < 0.05).

**Conclusions:**

Compared with N-TLIF, NM-TLIF has the advantages of being less invasive, yielding similar or better screw placement accuracy and achieving better symptom relief in the midterm postoperative recovery period. However, more attention should be given to real-time adjustment for pedicle insertion in NM-TLIF rather than just following the entry point and trajectory of the intraoperative plan.

## Background

Transforaminal lumbar interbody fusion (TLIF) is an effective and widely accepted treatment for lumbar degenerative diseases [1]. However, conventional TLIF inevitably leads to a large incision, paravertebral muscle atrophy, massive blood loss, significant postoperative pain, and a long recovery time [2].

Compared with TLIF, minimally invasive TLIF (MIS-TLIF) has been demonstrated to have significant advantages, including a smaller incision, less severe paravertebral muscle dissection, less blood loss, less severe postoperative pain, and faster postoperative recovery [3, 4], and to yield comparable clinical results [5, 6]. Nevertheless, due to the inadequate exposure of anatomical landmarks, traditional MIS-TLIF is sometimes associated with insufficient decompression, the inaccurate placement of cages, and an increased risk of pedicle screw malpositioning or pedicle perforation [7]. Moreover, multiple fluoroscopies are required to ensure accurate pedicle screw placement, which could increase the radiation exposure of patients and medical staff [8].

Following the introduction of cone-beam computed tomography (CT)–guided spinal navigation, numerous reports in the literature have demonstrated its utility in increasing the accuracy of pedicle screw placement and decreasing the incidence of neurological injury from pedicle screws misplacement [9, 10]. It also reduces the health risks associated with repeated fluoroscopy [11].

**Fig. 1:**
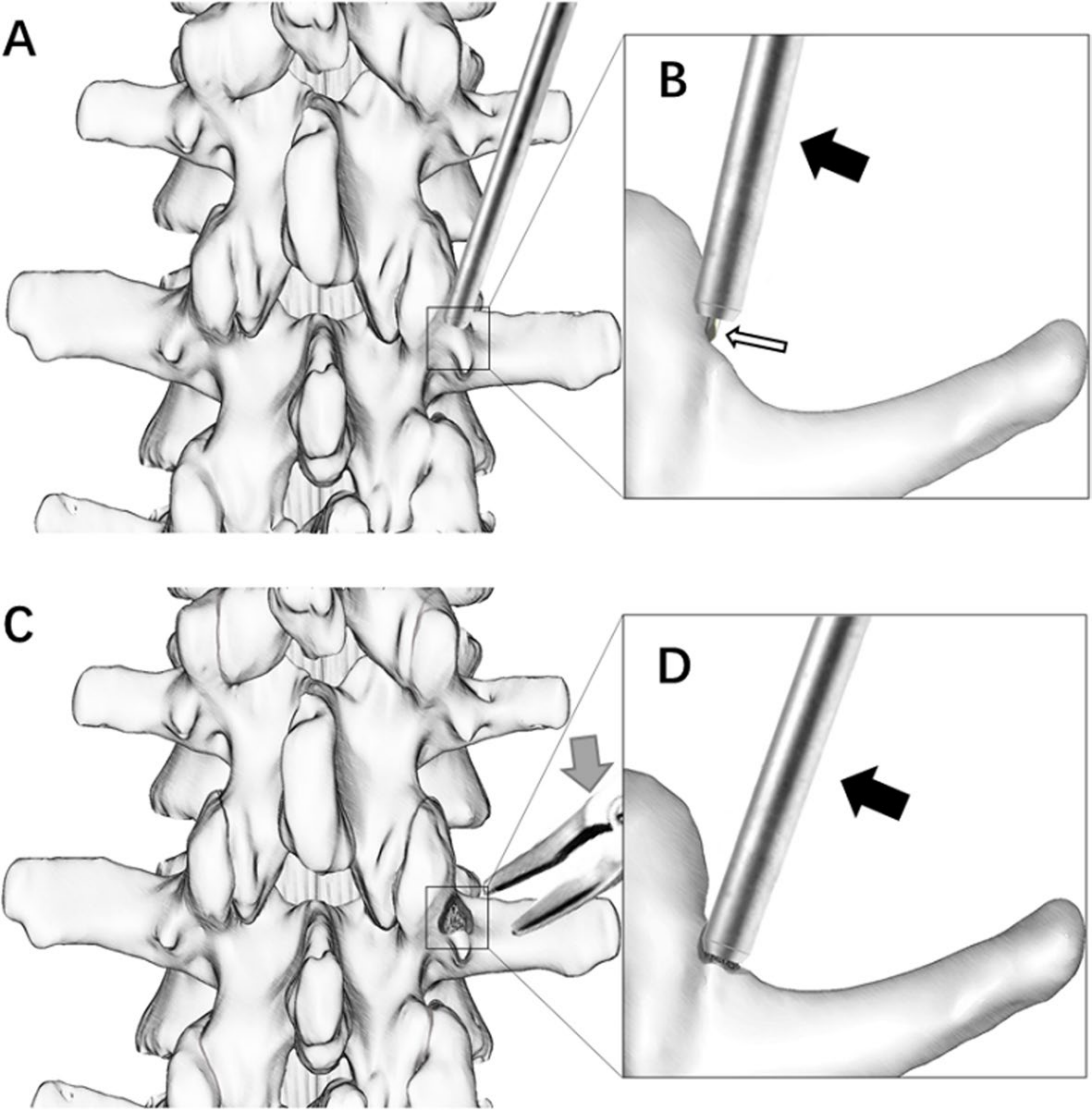
Difference between the two methods of pedicle screw implantation. **A** Posterior view of the spine. The cortical bone could not be excised under minimally invasive surgery, and an NDG was placed in the planned ideal insertion location and was drilled with a Kirschner wire to obtain the trajectory. **B** Axial view of the spine. The NDG at the ideal insertion location is on the inclined plane of the cortical bone. **C** Posterior view of the spine. In open surgery, the exact insertion point can be obtained by removing a piece of cortical bone with a rongeur. **D** Axial view of the spine. After the removal of the cortical bone, the NDG can be firmly anchored to the obtained relatively flat surface. Black arrow: navigation drill guide, NDG; grey arrow: rongeur; white arrow: Kirschner wire

With the use of navigation, the MIS-TLIF operation is more accurate, efficient, and safe [10]. However, in navigation-assisted MIS-TLIF (NM-TLIF), due to inadequate surrounding tissue dissection during the operation, the bone surface of the area in which the pedicle screw is placed is often uneven. The screw placement apparatus (e.g., the navigation drill guide, NDG) for NM-TLIF often cannot be fixed very well, which inevitably results in displacement of both the puncture point and the trajectory. This issue may increase the risk of pedicle screw misplacement or pedicle perforation, resulting in surgical failure. In contrast, the tissues surrounding the navigation-assisted TLIF (N-TLIF) area are fully exposed, and a definite screw setting point can be prepared by a rongeur to prevent the above issues to a certain extent (Fig. 1). Whether the effect of pedicle screw placement specifically in N-TLIF is better than that in NM-TLIF and yields better clinical efficacy has never been discussed. Therefore, the purpose of this study was to compare the pedicle screw accuracy and clinical outcomes between NM-TLIF and N-TLIF under O-arm navigation guidance to determine which surgical method is better and guide clinical decision making.

## Methods

### Patient selection

This study was approved by the ethics committee of the Third Military Medical University. The medical records of patients who underwent single-level posterior lumbar interbody fusion guided by O-arm-based navigation between May 2015 and May 2019 were selected. The study was based on a retrospective analysis of prospectively collected data. A noninferiority design was used in this study. The sample size was calculated based on previous studies that reported an accuracy rate of 83 to 99% for CT-based navigation-assisted pedicle screw implantation [12]. We assigned an accuracy rate of 97%, a noninferiority difference of − 7%, an approximate ratio of control to study subjects of 1, and a sample size of 102 pedicle screws per group, yielding a power of 90% and α of < 0.05. The patients were divided into the N-TLIF group and NM-TLIF group according to whether a minimally invasive or an open procedure was implemented during the operation. The inclusion criteria of this study were complaints of low back pain; varying degrees of radicular pain and neurological symptoms; single-level lumbar spinal stenosis, lumbar disc herniation, lumbar instability or spondylolisthesis, as demonstrated by anteroposterior, lateral, oblique, and flexion-extension plain radiographs, CT scans, and magnetic resonance imaging (MRI) scans; and a lack of response to extensive conservative therapy for at least 3 months before surgery. The exclusion criteria included trauma, active infections, tumors, spinal deformities, multisegmental fusion, a history of fusion, and cauda equina syndrome. All operations were performed by the same team of senior doctors. The clinical data of all patients were obtained from medical records and telephone follow-ups.

### Clinical evaluation

Patient age, gender, body mass index (BMI), American Society of Anesthesiologists (ASA) class, preoperative diagnoses, operative level, incision length, operative time, intraoperative blood loss, drainage volume, time to ambulation, analgesic use rate, blood transfusion rate, hospitalization duration, hospitalization cost and complications were assessed and recorded. Follow-ups, including clinical evaluations, were performed preoperatively and 1, 6, and 12 months after surgery. The minimum follow-up time was 12 months. The visual analog scale (VAS) score was used to evaluate pain in the lower back and lower extremities. The Oswestry disability index (ODI) was used to evaluate patients’ daily life functions.

### Radiological evaluations

Clinical qualitative accuracy was determined after the drainage tube was removed on the second day post-surgery using anterior-posterior and transverse flexural and telescopic ordinary X-ray and CT. The position of the pedicle screws was evaluated by postoperative CT scans in all cases. The screw position was graded from 0 to 3, with Grade 0 assigned to screws that did not perforate the pedicle. For the screws that did breach, Grade 1 was assigned to minor breaches of less than 2 mm, Grade 2 was assigned to breaches between 2 and 4 mm, and Grade 3 was assigned to breaches greater than 4 mm [13].

The absolute quantitative accuracy of the placement of each pedicle screw was measured using the method reported by Guha D., et al [14] The final screw position on postoperative CT was compared with the screenshot of the ideal screw entry point and trajectory planned by the intraoperative navigation system. Translational and angular deviations from the planned entry point and trajectory were measured in the axial and sagittal planes. In the axial plane, positive translational deviations denote a lateral deflection of the entry point, and positive angular deviations denote a more lateral trajectory. In the sagittal plane, positive translational deviations denote a superior deflection of the entry point, and positive angular deviations denote a more cranial trajectory. All image processing and measurements were performed using Mimics software (version 21, Materialise, Belgium).

MRI scans obtained one-year post-surgery were previously been used to evaluate multifidus muscle injury at the surgical site [15]. Multifidus muscle injury was assessed using a Picture Archiving and Communication System (PACS) workstation. In the axial images, T2-weighted MRI grayscale values of the psoas major and multifidus muscles were measured in a 300-mm^2^ circular region of interest. T2-weighted MRI grayscale values at the surgical segment and adjacent segments were measured, and their mean values represented the mean signal intensity of the psoas major and multifidus muscle, respectively. The T2-weighted signal intensity ratio was determined by dividing the mean signal intensity of the psoas major by the mean signal intensity of the multifidus muscle. The assessment of the images and measurements of the screw position were performed by a surgeon who was not involved in the study.

### Surgical procedure

#### NM-TLIF procedure

**Fig. 2:**
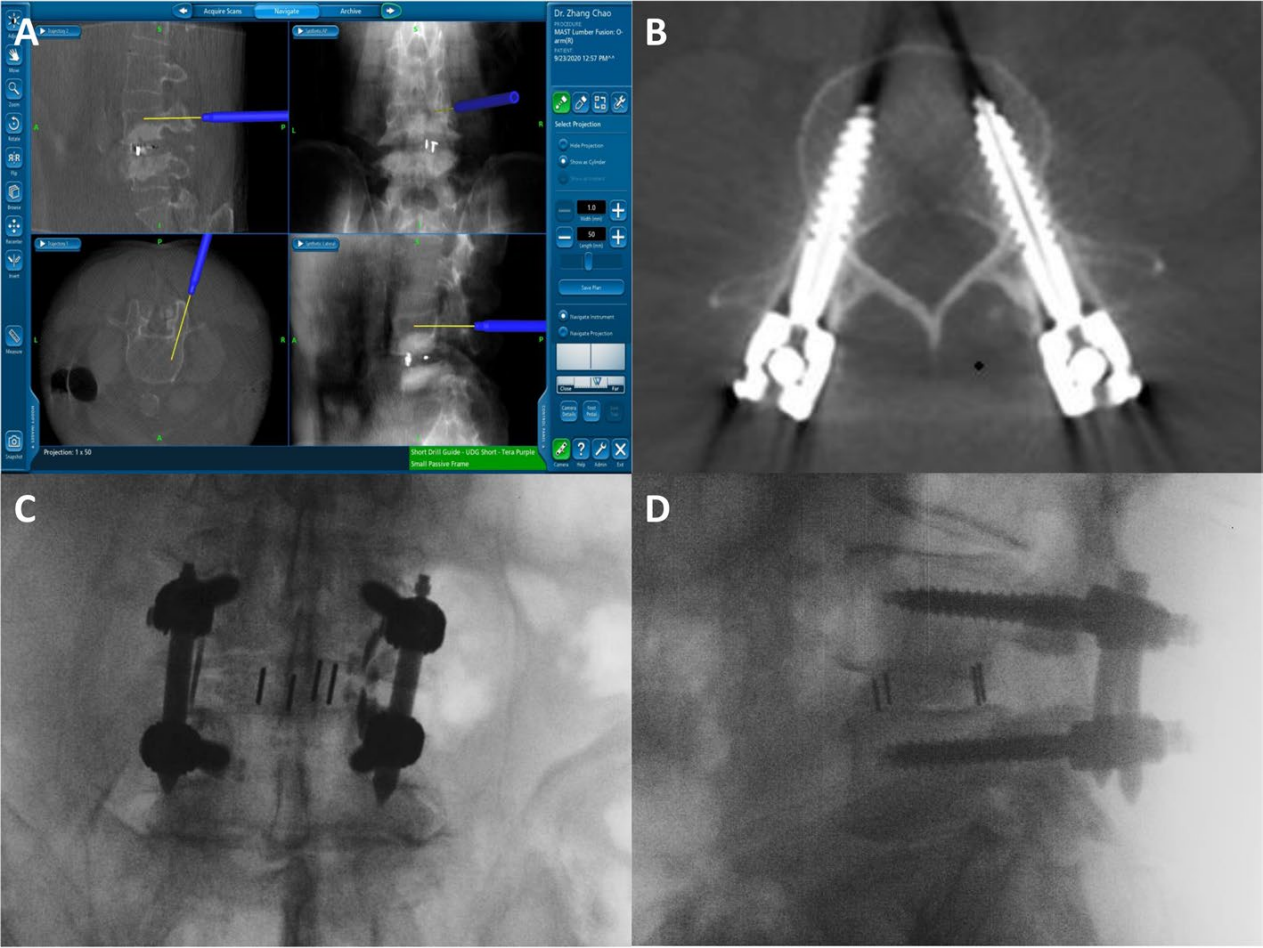
Intraoperative planning and outcomes. **A** A screenshot of the intraoperative navigation plan, showing the sagittal and axial views of the planned insertion trajectory and the positive and lateral views of the reconstructed fluoroscopy. **B** Postoperative CT reconstruction showing that the pedicle screws are completely within the vertebral pedicle. **C** A typical anterior view of a navigation-guided pedicle screw implant. **D** A typical lateral view of a navigation-guided pedicle screw implant

After general anesthesia was established, the patient was positioned prone on the operating Table. A 3.5- to 4-cm longitudinal incision was made 3 cm lateral to the midline on the decompression side for placement of an extensible retractor (Mast Quadrant Retractor System). The facet joints corresponding to the target intervertebral space were exposed. The entire facet joint and ligamentum flavum were removed to expose the nerve roots and dural sac. The adhered nerve roots were separated, and the nerve root canal was decompressed. Then, a complete discectomy was performed. The autogenous bone obtained during decompression was placed in the prepared intervertebral disc space, and a cage filled with bone fragments was placed obliquely. Two Kirschner wires were implanted into the posterior superior iliac spine of the patient, to which a reference frame was fixed. CT images of the surgical area were obtained by the O-arm and then transferred to the host navigation system (StealthStation S7 Surgical Navigation System, Medtronic, Minneapolis, Minnesota, USA) by a network cable for intraoperative three-dimensional (3D) reconstruction. After the reconstructed image was obtained, the instrument was registered, and the accuracy of navigation was initially assessed by placing the probe on the spinous process and observing whether the image corresponded to the navigation image. The same pedicle screw placement technique was used on the decompression and the opposite sides. The entry point and trajectory of the pedicle screw were planned on the sagittal and axial views of the navigation system, and a screenshot of the plan was recorded. A guidewire was placed with an electric drill under the guidance of an NDG (Fig. 2). After implantation of all four guide wires, fluoroscopy was used to verify the consistency between the guide wires and the intraoperative plan. After 4 pedicle screws were inserted along the verified guidewire, the accuracy of screw placement was finally verified by fluoroscopy. Then, titanium rods were inserted to connect the pedicle screws. The intervertebral space was pressurized, and the nut was tightened.

#### N-TLIF procedure

After general anesthesia was established, the patient was placed in the prone position, and a median incision of approximately 8 cm was made with the target intervertebral space as the center. The paravertebral muscles were stripped, and the spinous process of the upper vertebral body was exposed and fixed with the spinous process reference frame. O-arm scanning was performed to reconstruct the image of the operative area. Pedicle screws were implanted under the assistance of O-arm navigation. After the entry point and trajectory were planned and the screenshot was saved, some bone cortex was removed with a rongeur at the planned position to make space for the definite insertion point. The subsequent implantation process was consistent with that performed for NM-TLIF, and the position was confirmed by fluoroscopy. Intervertebral space distraction was performed after bilateral titanium rod implantation. Target space laminectomy decompression, nerve root release, intervertebral space treatment and bone graft fusion were performed. Fluoroscopy confirmed that the location of the cage was ideal. The position of the connecting rod was properly adjusted, and the nut was properly pressurized and locked.

### Statistical analyses

Statistical analyses were performed with SPSS (version 23.0, SPSS Inc., Chicago, IL, USA). Continuous variables were compared between the groups by Student’s t test or the two-sample Mann-Whitney U test. Categorical variables were compared between the two groups by the chi-square test or Fisher’s exact test. The ODI and VAS scores were analyzed by repeated-measures ANOVA. *P* < 0.05 was considered statistically significant.

## Results

### Patients characteristics

The data from 28 patients who underwent NM-TLIF and 26 patients who underwent N-TLIF were included in the analysis, and all patients were treated with single-segment pedicle screw fixation guided by O-arm-based navigation. The demographic characteristics were comparable between the two groups; there were no significant differences in age, gender, BMI, ASA classification or preoperative diagnosis between the two groups. All patients underwent surgery for degenerative diseases (spondylolisthesis, lumbar instability, lumbar spinal stenosis or lumbar disc herniation) and were followed up for at least 12 months (Table 1).

**Table 1:** Patients characteristics

	NM-TLIF (*n* = 28)	N-TLIF (*n* = 26)	*P* Value
**Demographics**			
Age (years)	52.1 ± 12.1	54.5 ± 13.7	0.525
Female gender^a^	16 (57.1%)	15 (57.7%)	0.967
Body mass index (kg/m^2^, BMI)	23.0 ± 2.6	24.0 ± 2.7	0.663
ASA class ^c^	1.2 ± 0.42	1.3 ± 0.47	0.438
**Diagnosis**			
Spondylolisthesis^a^	12 (42.9%)	14 (53.8%)	0.419
Lumbar instability	10 (35.7%)	6 (23.1%)	0.310
Lumbar spinal stenosis^b^	4 (14.3%)	5 (19.2%)	0.626
Lumbar disc herniation^a^	2 (7.1%)	1 (3.8%)	0.597
**Follow up (months)**			
Range	13–59	12–59	
Mean	39.5 ± 6.1	37.0 ± 6.7	0.544

^a^Chi-squared test. ^b^Fisher’s exact test. ^c^Two-Sample Mann–Whitney U Test. Otherwise, an independent-samples *t* test was performed with equal variances assumed

### Clinical evaluations

There were no statistically significant differences between the two groups with respect to the operative level, operative time, hospitalization duration or operative cost. The incision length was significantly shorter in the NM-TLIF group than that in the N-TLIF group (*P* < 0.05), the intraoperative blood loss and postoperative drainage volume were significantly lower in the NM-TLIF group than in the N-TLIF group (*P* < 0.05), and the drainage tube was pulled out approximately 1 day later in the N-TLIF group than in the NM-TLIF group (*P* < 0.05). Four patients in the N-TLIF group (15.4%) and none in the NM-TLIF group required blood transfusion. The postoperative analgesia rate was also significantly higher in the N-TLIF group (*P* < 0.05) (Table 2), indirectly reflecting that the postoperative incision pain in the N-TLIF group was greater than that in the NM-TLIF group.

**Table 2:** Surgical data

	MN-TLIF (*n* = 28)	N-TLIF (*n* = 26)	*P* Value
Overall parameters			
**Operative level,** ***n*** **(%)**			
L3-L4^b^	1 (3.6%)	2 (7.7%)	0.604
L4-L5^a^	18 (64.3%)	17 (65.4%)	0.933
L5-S1^a^	9 (32.1%)	7 (26.9%)	0.675
**Perioperative indicators**			
Incision lengths (cm)^c^	4.1 ± 1.2	7.8 ± 1.0	< 0.001
Intraoperative blood loss (ml) (M ± IQR) ^c^	150.0 ± 217.5	200.0 ± 150.0	0.017
Operative time (min)	193.8 ± 57.9	195.0 ± 56.9	0.558
Drainage volume (ml)^c^	64.6 ± 65.8	186.8 ± 150.0	< 0.001
Time to ambulation (day)^c^	2.1 ± 0.3	3.2 ± 1.3	< 0.001
Hospitalization duration (day)^c^	6.1 ± 2.8	8.5 ± 3.3	0.002
Blood transfusion rate^d^	0 (0%)	4 (15.4%)	
Analgesia ratio^a^	3 (10.7%)	12 (46.2%)	0.004
Hospitalization cost (CNY)^c^	61,677.6 ± 32,991.7	72,397.1 ± 20,184.2	0.665

^a^Chi-squared test. ^b^Fisher’s exact test. ^c^Two-sample Mann-Whitney U Test. Otherwise, an independent-samples *t* test was performed with equal variances assumed. ^d^In 4 cases, intraoperative blood loss was greater than 800 ml, up to 1200 ml, and the hemoglobin concentration in all cases was less than 70 g/L, which met the criteria for a blood transfusion

There were no significant differences in the ODI, low back pain VAS score or lower limb pain VAS score between the two groups before surgery (*P* > 0.05). These indicators showed a gradually decreasing trend in the two groups postoperatively. The ODI and low back pain VAS score were significantly lower in the NM-TLIF group at 1 and 6 months postoperatively (*P* < 0.05). There were no significant differences in the VAS score for lower extremity pain postoperatively (*P* > 0.05). Additionally, there was no significant difference between the two groups in the three indicators at the final follow-up (*P* > 0.05) (Table 3). In all cases, no instrument-related complications (such as nerve root injury, cerebrospinal fluid leakage, and visceral injury) were observed, and revision surgery was not required due to pedicle screw malpositioning.

**Table 3:** Comparison of clinical parameters between the two groups

	NM-TLIF (*n* = 28)	N-TLIF (*n* = 26)	*P* Value
**ODI scores**			0.121
Preoperative	49.4 ± 4.7	50.3 ± 5.9	0.548
Postoperative 1 month	20.5 ± 6.7	25.7 ± 8.2	0.013
Postoperative 6 months	16.1 ± 5.9	19.8 ± 6.9	0.040
Postoperative 12 months	12.8 ± 5.7	14.0 ± 6.1	0.478
Final follow-up	8.0 ± 5.2	9.8 ± 5.4	0.345
**Low back pain VAS scores**			0.006
Preoperative	6.9 ± 1.0	7.3 ± 1.3	0.200
Postoperative 1 month	2.9 ± 1.0	3.6 ± 0.8	0.006
Postoperative 6 months	1.6 ± 0.8	2.3 ± 0.9	0.006
Postoperative 12 months	1.2 ± 0.9	1.6 ± 0.9	0.094
Final follow-up	0.7 ± 0.7	0.9 ± 0.8	0.384
**Lower extremity pain VAS scores**			0.865
Preoperative	5.3 ± 1.7	5.9 ± 1.6	0.211
Postoperative 1 month	1.5 ± 0.9	1.4 ± 0.7	0.733
Postoperative 6 months	1.4 ± 1.0	1.2 ± 1.2	0.361
Postoperative 12 months	1.1 ± 0.9	1.0 ± 1.0	0.672
Final follow-up	0.3 ± 0.5	0.4 ± 0.6	0.660

ANOVA for repeated design data

### Radiological evaluations

In this study, 216 pedicle screws were graded based on postoperative CT images; 112 of the screws were implanted in the NM-TLIF group, and 104 were implanted in the N-TLIF group. The clinical qualitative accuracy rate (Grades 0 and 1) in the two groups was 97.3% (NM-TLIF) and 96.2% (N-TLIF). Three screws in the NM-TLIF group and 4 screws in the N-TLIF group were not aligned (Grade 2), but none of the patients showed symptoms of spinal cord or nerve injury. No instances of Grade 3 pedicle screw placement were observed. There was no significant difference in the accuracy rate between the two groups (*P* > 0.05) (Table 4).

**Table 4:** Clinical quality accuracy of pedicle screws

Level treated	Grade 0	Grade 1	Grade 2	Grade 3	Accuracy rate (Grade 0 and 1)
NM-TLIF (*n* = 112)					
L3	2				2 (100%)
L4	35	2	1		37 (97.4%)
L5	48	4	2		52 (96.3%)
S1	15	3			18 (100%)
Total	100	9	3		109 (97.3%)*
N-TLIF (*n* = 104)					
L3	3	1			4 (100%)
L4	36	1	1		37 (97.4%)
L5	43	3	2		46 (95.8%)
S1	12	1	1		13 (92.9%)
Total	94	6	4		100 (96.2%)*
*P* Value*					0.713

*Fisher’s exact test

The absolute quantitative accuracy results showed that the axial translational error, sagittal translational error and sagittal angle error in the NM-TLIF group were significantly greater than those in the N-TLIF group (*P* < 0.05). Compared with that in the N-TLIF group, the entry point in the NM-TLIF group was more prone to lateral deflection and inferior deflection, while the screws in the NM-TLIF group were more prone to exhibiting a more cranial trajectory. However, the axial angle error was not statistically significant (*P* > 0.05) (Table 5).

**Table 5:** Absolute quantitative accuracy of pedicle screw placement

	NM-TLIF (*n* = 112)	N-TLIF (*n* = 104)	*P* Value
Axial translational error (mm)	0.82 ± 2.77	−0.33 ± 0.76	< 0.001
Axial angular error (degree)	0.49 ± 5.62	0.14 ± 4.27	0.862
Sagittal translational error (mm)	−0.89 ± 2.04	0.16 ± 1.03	< 0.001
Sagittal angular error (degree)	1.34 ± 4.79	− 0.83 ± 3.12	< 0.001

Two-Sample Mann–Whitney U Test

**Fig. 3:**
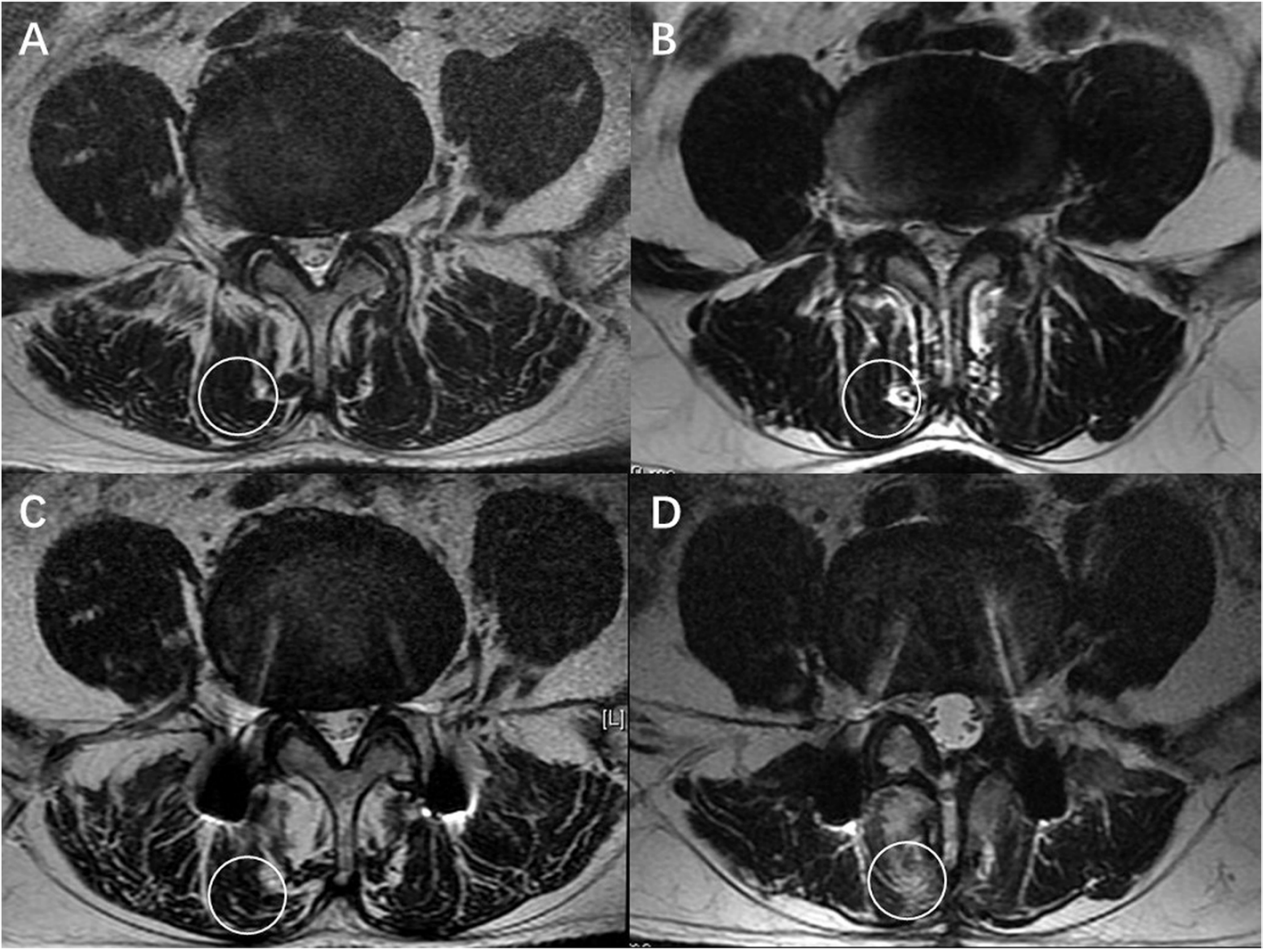
One-year follow-up MRI comparison. **A** + **C** MRI of a 57-year-old female preoperatively and at the one-year follow-up in the NM-TLIF group. **B** + **D** MRI of a 55-year-old female preoperatively and one-year follow-up in the N-TLIF group. Preoperative and one-year follow-up MRI images showing that the degree of multifidus atrophy in the NM-TLIF group was significantly lower than that in the N-TLIF group. Circle: region of interest, 300 mm^2^

**Fig. 4:**
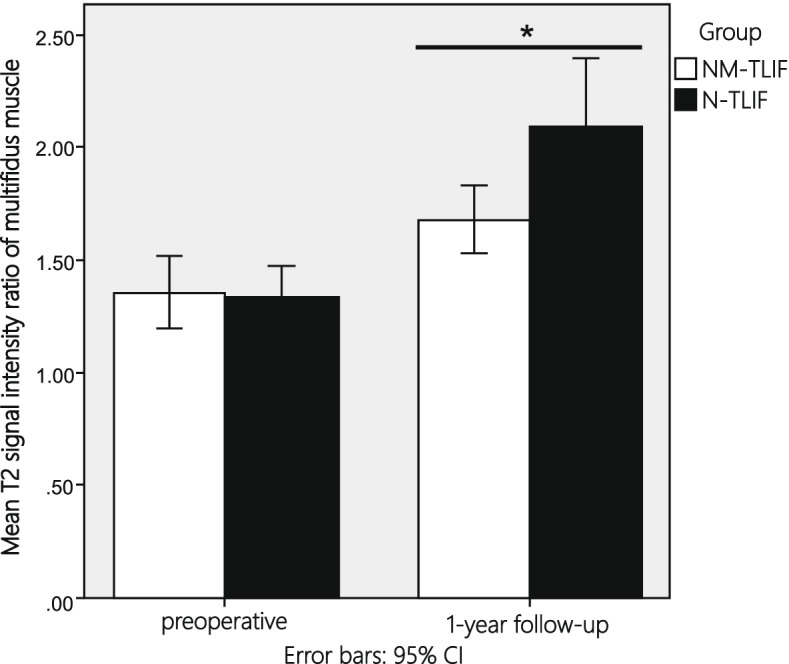
Mean T2-weighted MRI signal intensity ratio of the multifidus muscle. Bar graph showing preoperative and 1-year follow-up differences in the mean T2-weighted MRI intensity ratio of the multifidus muscle between the 2 groups. The mean T2-weighted MRI intensity ratio of the multifidus muscle in the NM-TLIF group was significantly lower than that in the N-TLIF group at the 1-year follow-up (*P* = 0.022). *P value < 0.05

One-year post-surgery, muscle atrophy and fat infiltration were observed in both groups (Fig. 3). The mean T2-weighted signal intensity of the multifidus muscle in the NM-TLIF group was significantly lower than that in the N-TLIF group (*P* < 0.05) (Fig. 4).

## Discussion

This retrospective cohort study revealed that N-TLIF does not yield better accuracy or clinical outcomes than NM-TLIF. In this study, the clinical results of 54 patients and the placement accuracy of 216 pedicle screws were retrospectively analyzed, and it was found that the two different surgical methods achieved excellent screw placement accuracy, with no significant differences between them. Both procedures can effectively alleviate patient symptoms and yield similar clinical outcomes.

Our study suggests that, regarding the clinical outcomes, within at least 12 months of follow-up, the postoperative lumbar pain VAS and lower extremity pain VAS scores and ODI improved significantly in the two groups. There were no significant differences in the intraoperative or postoperative complications between the two groups. Both open and minimally invasive procedures, with the assistance of navigation, yielded consistently good results. However, the use of intraoperative navigation increased the operative time over that reported in our previous studies at the same institution [16], which is consistent with the results of previous research [17]. According to the findings reported by other authors, extra surgical time is mainly required for the preparation of the navigation equipment, whereas the screw implant time is dramatically reduced; therefore, the operative process is improved with navigation [18, 19].

In our study, the two methods of surgery involving intraoperative O-arm navigation assistance yielded high accuracy, with no statistically significant difference between the methods (97.3% vs. 96.2%, *P* > 0.05). The results showed that the accuracy of NM-TLIF was equal to that of N-TLIF due to the inherent drawbacks of minimally invasive surgery, such as insufficient anatomical standard exposure, bone surface unevenness and ease of insertion point deviation, which is consistent with previous research results [20, 21]. Importantly, the absolute accuracy results showed that both axial and sagittal translational errors were greater in the NM-TLIF group than in the N-TLIF group, which verifies our hypothesis. However, despite the larger absolute accuracy error at the entry point in the NM-TLIF group, there was no statistically significant difference in the final accuracy of pedicle screw placement between the two groups. This result suggests that there is no direct correlation between pedicle screw placement accuracy and the absolute accuracy of entry point deflection, which is consistent with the results of a previous study [14]. Meanwhile, this result reveals that intraoperative navigation can provide surgeons with the ability to correctly adjust the screw orientation in the cases of insertion point deviation [20, 22]. Navigation-based visualization can aid not only in planning when using an NDG to identify the entry point, but also in adjusting the trajectory direction when an electric drill is used to obtain the trajectory. The surgeon can adjust the trajectory after the drill bit enters the bone cortex to achieve a good pedicle screw placement. We suggest that attention should be given to the screw orientation during the entire screw placement process in NM-TLIF and that adjustments should be made as needed.

In this study, N-TLIF did not yield better screw placement accuracy or better clinical outcomes. In contrast, NM-TLIF seemed to have more advantages, such as being less invasive, causing significantly less damage to the surrounding spinal muscle tissues, and facilitating better long-term muscle tissue recovery than N-TLIF. Serban, D. et al. [23] divided patients into a standard TLIF group and an MIS-TLIF group. The results showed that the magnitude of improvement in the ODI from before to after surgery was statistically significant in both groups and similar between groups. The two techniques yielded similar clinical and radiological outcomes at 1 year, while the patients undergoing MIS-TLIF had a shorter hospital stay. Their results are similar to ours and demonstrate the advantages of MIS-TLIF.

The main limitations of this study are as follows. First, this study was a retrospective study, with a low level of evidence and the possibility of selection bias. Prospective randomized controlled studies with larger sample sizes should be carried out in the future to overcome the limitations of existing studies. Second, we did not compare radiation exposure levels between the different surgical procedures. Previous studies reported only general assessments of relative radiation exposure differences between procedures, but absolute values were not available [24, 25]. Further studies of intraoperative radiation monitoring using specialized instruments are needed. Third, we used real O-arm CT positional data of patients obtained intraoperatively, but this inevitably resulted in an increase in the radiation exposure of patients. The use of preoperative CT and intraoperative X-ray registration may be an acceptable alternative to reducing patient radiation exposure, although this procedure may cause some registration errors.

## Conclusion

Navigation-guided percutaneous pedicle screw placement does not reduce the accuracy of pedicle screw implantation but could achieve the same screw placement accuracy as the open procedure. It should be continuously improved concerning its accuracy and stability. Compared with the N-TLIF group, the NM-TLIF group achieved comparable clinical results, with the additional minimally invasive benefits of a shorter incision length, less intraoperative blood loss, a smaller postoperative drainage volume, earlier ambulation time, a lower blood transfusion rate, a lower postoperative analgesia rate, and shorter hospital stay. It is essential to optimize the surgical process, enhance the efficiency of navigation surgery, shorten the operative time, reduce the cost of the navigation system, and expand the application scope of the navigation method. Moreover, NM-TLIF can even provide better symptom relief in the midterm postoperatively. However, more attention should be given to real-time adjustments during pedicle screw insertion in NM-TLIF surgery rather than just following the entry point and trajectory of the intraoperative plan.

## Data Availability

The datasets used and analyzed during the current study are available from the corresponding author on reasonable request.

## Abbreviations

ASA: American Society of Anesthesiologists
BMI: Body mass index
CT: Computed tomography
MIS-TLIF: Minimally invasive transforaminal lumbar interbody fusion
MRI: Magnetic resonance imaging
NM-TLIF: Navigation-assisted minimally invasive transforaminal lumbar interbody fusion
N-TLIF: Navigation-assisted transforaminal lumbar interbody fusion
NDG: Navigation drill guide
ODI: Oswestry disability index
TLIF: Transforaminal lumbar interbody fusion
VAS: Visual analog scale
3D: Three dimensional
